# A Bayesian Difference-in-Difference Framework for the Impact of Primary Care Redesign on Diabetes Outcomes

**DOI:** 10.1080/2330443X.2019.1626310

**Published:** 2019-07-18

**Authors:** James Normington, Eric Lock, Caroline Carlin, Kevin Peterson, Bradley Carlin

**Affiliations:** a Division of Biostatistics, School of Public Health, University of Minnesota, Minneapolis, MN; b Department of Family Medicine and Community Health, University of Minnesota, Minneapolis, MN; c Counterpoint Statistical Consulting, LLC, Minneapolis, MN

**Keywords:** Bayesian hierarchical modeling, Diabetes, Difference-in-differences, Errors in covariates, Patient-centered medical home, Primary care redesign

## Abstract

Although national measures of the quality of diabetes care delivery demonstrate improvement, progress has been slow. In 2008, the Minnesota legislature endorsed the patient-centered medical home (PCMH) as the preferred model for primary care redesign. In this work, we investigate the effect of PCMH-related clinic redesign and resources on diabetes outcomes from 2008 to 2012 among Minnesota clinics certified as PCMHs by 2011 by using a Bayesian framework for a continuous difference-in-differences model. Data from the Physician Practice Connections-Research Survey were used to assess a clinic’s maturity in primary care transformation, and diabetes outcomes were obtained from the MN Community Measurement (MNCM) program. These data have several characteristics that must be carefully considered from a modeling perspective, including the inability to match patients over time, the potential for dynamic confounding, and the hierarchical structure of clinics. An ad-hoc analysis suggests a significant correlation between PCMH-related clinic redesign and resources on diabetes outcomes; however, this effect is not detected after properly accounting for different sources of variability and confounding. Supplementary materials for this article are available online.

## 1. Introduction

Over the last 10 years, national “quality of care” measures have demonstrated that important gaps exist in the delivery of optimal diabetes care (HEDIS 2018). Primary care practices provide an important focus for improving medical care for individuals with diabetes and other chronic diseases (Blackwell, Lucas, and Clarke 2014; Berwick, Nolan, and Whittington 2008). New models of care delivery that will increase coordination, emphasize prevention, and enhance collaboration between multidisciplinary teams have been proposed to increase the effectiveness of primary care delivery (Richardson et al. 2001). In 2008, the Minnesota state legislature endorsed the patient-centered medical home (PCMH) as the preferred model for primary care redesign (Minnesota Statutes 2008). State certification as a PCMH requires satisfactory provision of a comprehensive set of resources and services validated through onsite state inspection, annual reporting, and regular tracking. This legislated process provided the setting for a natural experiment to compare the delivery of standardized and validated clinical services on diabetes outcomes over the last 10 years.

Our goal is to investigate the effect of PCMH-associated clinical services and resources on diabetes outcomes among patients at Minnesota clinics certified as PCMHs by 2011. Factors that influence diabetes outcomes are complex and interrelated. In addition, factors influencing a clinic’s decision about which services and resources to implement may be correlated with diabetes outcomes, making causal inference challenging. A cross-sectional analysis approach is therefore limited, even with propensity adjustment (Rosenbaum and Rubin 1983) or other techniques, because of the potential for unmeasured confounding. Instead, we use data from before and after PCMH certification to assess the association between changes in a composite measure of clinic services and changes in diabetes outcomes, to account for confounding from static clinic characteristics.

By focusing on differences in change over time, our approach can be considered a *difference-in-differences* (DiD) approach. DiD is a popular method for estimating effects of policy interventions and changes, that do not affect every entity at the same time and in the same way (Lechner 2010). Under a classical DiD framework, we estimate the effect of a policy by computing the observed difference between how the “treated” group changes and how the “control” group changes: $\hat{\Delta }\equiv {\hat{\delta }}_{\text{Trt}}-{\hat{\delta }}_{\text{Ctrl}}=({\bar{y}}_{\text{Trt,Post}}-{\bar{y}}_{\text{Trt,Pre}})-({\bar{y}}_{\text{Ctrl,Post}}-{\bar{y}}_{\text{Ctrl,Pre}})$. DiD models avoid the effect of static confounders that may affect a simple comparison of post-treatment outcomes between treated and control groups. As a canonical example of the classical difference-in-difference model, Card and Krueger (1994) compared the change in employment in New Jersey versus Pennsylvania after New Jersey adopted an increase in the minimum wage; in this context New Jersey could be considered the treated condition and Pennsylvania the control condition. In our context, the primary predictor is a continuous measure of clinic services rather than a binary measure of treatment status, and so we instead focus on assessing the association between change in outcome and change in predictors. We call this a continuous difference-in-difference (CDiD) model; as an example, Card (1992) considered the association between the change in minimum wage and the change in employment across several states. In this article, we offer a Bayesian approach for the CDiD model, which provides a natural framework to accommodate the hierarchical data structure and other sources of variation in the present application to PCMH effects on diabetes outcomes.

These data have several characteristics that must be carefully considered from a modeling perspective, including the following:

1. *Inability to match patients*: To protect patient identities, the data were formatted so that we were unable to match patient records across years. Thus, our model measures the relationship between maturity in primary care transformation and mean change in diabetes outcomes, averaged across patients at the *clinic-level*. However, the variability of the mean difference in clinic outcomes and covariates can differ depending on the number of patients observed at a clinic and the heterogeneity of the patient population within a clinic. We must account for this clinic-level heteroscedasticity.
2. *Dynamic confounding*: Although the CDiD approach inherently balances static confounders, it is possible that changes in mean diabetes outcomes are driven by clinic-level changes. We discuss how to adjust for observable clinic-level changes in demographic distributions, such as sex, age, and type of insurance used.
3. *Hierarchical structure*: The outcome and predictors are hierarchical, as patients visit clinics which exist within health care systems. Clinics that belong to the same system organization likely have similar policies and electronic health record systems, and may also be similar geographically, economically, and demographically. It is unreasonable to assume mean diabetes outcomes across clinics within a system are uncorrelated, so we use a Bayesian hierarchical model as a natural way to account for this structure, and extend our model to include system organization-level effects.

We discuss these and other modeling considerations in technical detail in Section 4.

Section 2 presents notation and describes the data used in this study. Section 3 describes a simple ad-hoc analysis of these data, which results in a misleading conclusion. Section 4 proposes a hierarchical model to evaluate how a clinic’s maturity in primary care transformation relates to a change in diabetes-related outcomes, and Section 5 describes the prior specification in a Bayesian framework. Section 6 presents the Markov chain Monte Carlo (MCMC) procedure used to approximate the posterior distribution. Section 7 presents the results of our analysis, Section 8 describes simulation studies justifying important aspects of our model, and Section 9 offers discussion, suggests limitations, and concludes the article.

## 2. Data and Notation

In what follows, non-bolded quantities represent scalars while bolded quantities represent vectors or matrices. Tables 1 and 2 present the Greek and Roman notation used throughout, respectively. The reader should reference these tables as we present the model and results.

Section 2.1 describes the patient-year level data available to model diabetes outcomes. Section 2.2 describes data used to capture the characteristics of each patient’s neighborhood. Section 2.3.1 describes a survey that captures specific details about clinic structure and programs available, and Section 2.3.2 describes how we summarize these clinic characteristics into a measure of maturity in primary care transformation.

### 2.1. MN Community Measurement Optimal Diabetes Care

The MN Community Measurement (MNCM) Optimal Diabetes Care dataset contains the primary measures of optimal diabetes care incorporated into the MNCM “D5” standard of care for patients living with diabetes. The five criteria that are included in the 2012 MNCM D5 measure include low-density lipoprotein (LDL) below 100 mg/dL, blood pressure below 140/90 mmHg, hemoglobin A1c below 8%, daily use of aspirin or other antiplatelet medication if diagnosed with ischemic vascular disease (IVD), and documentation of non-smoking status. The diabetes outcome ***Y*^(yr)^** to be analyzed from MNCM is a vector of clinic average A1c, LDL, and systolic blood pressure (SBP). Patient-year records containing invalid measurement dates or values for these outcomes were omitted from the averages. The majority of the removed records (cumulatively, 6.3%) were due to missing or invalid dates associated with the A1c (5.3%) or LDL (3.1%) measurements. In addition, we excluded a few patient-years with values outside the range 3 and 25% for A1c scores (0.3%), 0 and 1000 mg/dL for LDL scores (1.2%), and excluded SBP scores that were less than 50 mmHg or less than the diastolic blood pressure (<0.1%). Available covariates at the patient level include insurance type (e.g., Commercial, Medicare, Medicaid), age, sex, and indicators of whether or not patient was diagnosed with IVD, and Type 1 or 2 diabetes. Depression diagnosis indicators were not available until 2010, so that covariate was excluded from the analysis. We were interested in measuring improvements in Type 2 diabetes, so patients identified as having Type 1 diabetes were also excluded from the analysis. Average demographic characteristics at the clinic level are used as covariates in our analysis.

### 2.2. American Community Survey

The American Community Survey (ACS) is an annual survey conducted by the U.S. Census Bureau which administers a questionnaire to a sample of addresses capturing many of the variables included in the long form decennial census. We use the survey results aggregated to the ZIP code summary level, matched to patient ZIP code to describe the environment in which the patient lives and functions. Using confirmatory factor analysis, Swaney (2018) computes measures of socioeconomic status in the patients’ neighborhoods (income and wealth) as a function of education, housing costs, use of the Supplemental Nutritional Assistance Program (SNAP), household income, and family structure. We also capture the influence of race and ethnicity by including the percentage of the ZIP code residents who are white, non-Hispanic. These three patient-level residential characteristics are averaged within clinic and included in our analysis.

### 2.3. Physician Practice Connections-Research Survey (PPCRS) and Clinic Scores

#### 2.3.1. The Survey

The Physician Practice Connections-Research Survey (PPCRS) (Solberg et al. 2013) is a survey designed to measure primary care organizational infrastructure across multiple domains, grounded in five of the six domains of Bodenheimer and Wagner’s chronic care model (CCM) (Bodenheimer, Wagner, and Grumbach 2002): health care organization, delivery system redesign, clinical information systems, decision support, and self-management support. Each question on the PPCRS in turn corresponds to one of these five CCM domains. The PPCRS was sent to all Minnesota primary care sites that had been certified as health care homes (Minnesota’s version of a PCMH) in 2011. Respondents were asked to report organizational structure at present (2011) and (by recall) in 2008.

#### 2.3.2. Clinic Scores

Only clinics certified by the state of Minnesota as a health care home within the first year of the health care home legislation’s effective date were sent the PPCRS survey for completion, so our population represents a pool of “early adopter” clinics. The PPCRS responses are used to infer a continuous metric of how advanced the clinic is in transformation of primary care delivery. In particular, the “clinic score” (*c_sj_*) is defined as the score from the first principal component (Jolliffe 1986), that is, the corresponding element of the first left-singular vector from the singular value decomposition of the row-centered survey matrix. We represent the *change* in a clinic’s maturity in primary care transformation as $c_{\mathrm{sj}}^{\text{diff}}$, the change in clinic score from 2008 to 2011: $c_{\mathrm{sj}}^{\text{diff}}\equiv c_{\mathrm{sj}}^{\left(2011\right)}-c_{\mathrm{sj}}^{\left(2008\right)}$. Clinics were de-identified, so their corresponding scores were uninterpretable at face value. However, the clinic score had a positive correlation with almost all questions in the PPCRS survey, and positive responses tend to represent a more mature transformation in primary care redesign. A simple histogram of ***c*^diff^** revealed that all but one clinic score change was positive, implying that higher clinic score corresponds to a more mature primary care transformation. Moreover, an analysis of the PPCRS data using Joint and Individual Variation Explained (JIVE) (Lock et al. 2013) only identified one latent component that was present across all CCM domains, and this component was closely correlated to the first principal component; this suggests that other structure in the PPCRS data is more granular. Further clustering analyses based on the Hamming distance (Hamming 1950) yielded only two groups of clinics, which can be thought of as having “more mature clinic transformation” and “less mature clinic transformation”.

## 3. An Ad-Hoc Analysis

**Figure 1.**
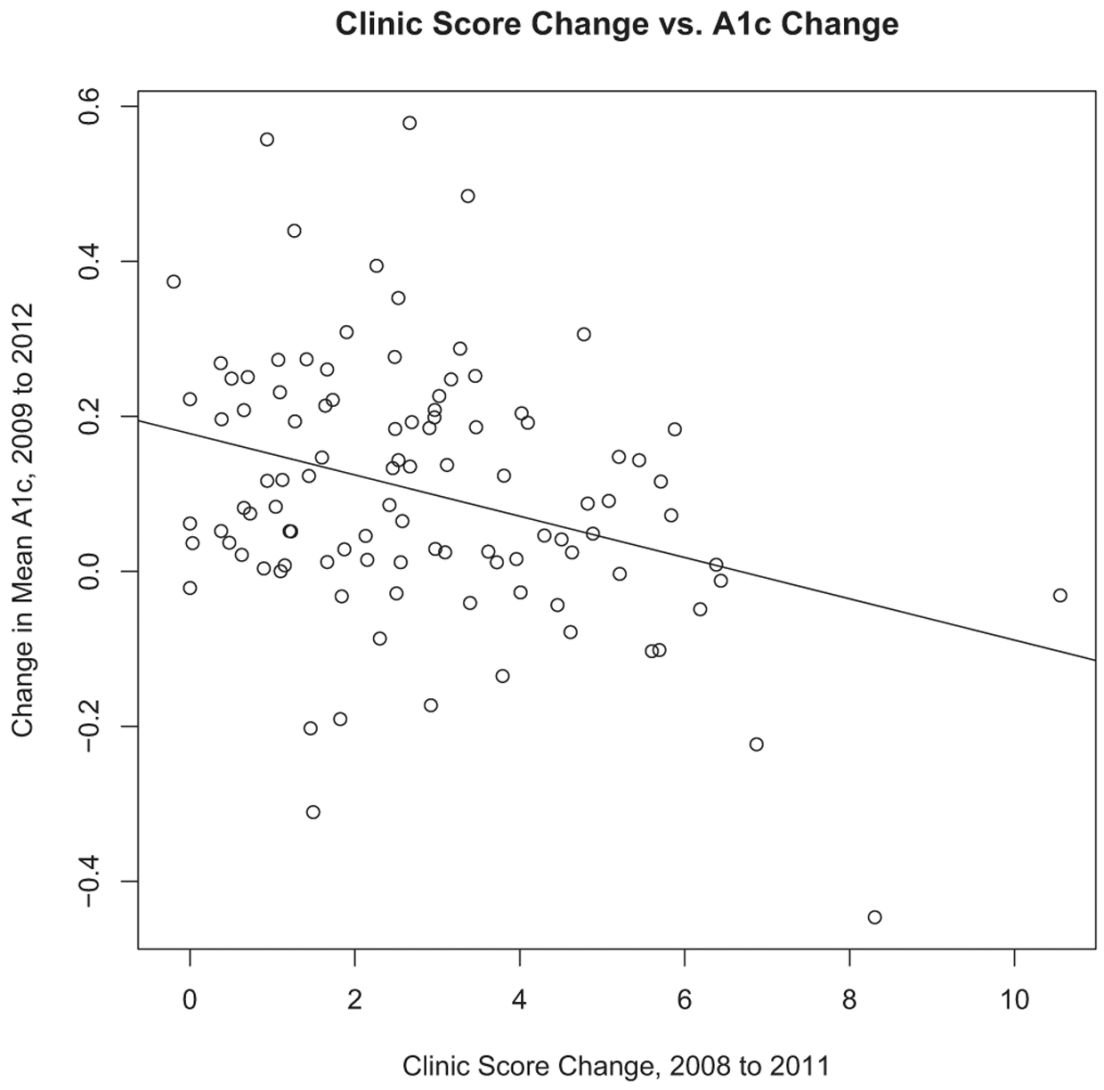
Changes in clinic score by changes in mean A1c with simple linear regression line.

A simple correlation analysis between mean change in A1c score and ***c*^diff^** across clinics yields a significant negative correlation $\hat{\rho }=-0.318$ (*p*-value = 0.001). Figure 1 illustrates the marginal bivariate relationship between a clinic’s change in mean A1c score and change in clinic score with its simple linear regression line. This suggests that a clinic that has made greater strides in primary care redesign observes improved mean diabetes outcomes (lower A1c is a desirable diabetes outcome). However, this result considers only the marginal relationship between the the two variables, and fails to account for several important aspects, including (1) the hierarchical structure of the data, (2) potential confounders in the form of shifting clinic demographics, (3) the variability and heteroscedasticity of the outcome and covariates resulting from different clinic-year sample sizes, and (4) confounding between ***c*^diff^** and baseline clinic score ***c***^**(2008)**^. As shown in Section 7, a causal link between the change in clinic score and change in our outcome measures (including A1c score) is much less clear after accounting for these features.

## 4. The Model

Here we describe a comprehensive Bayesian model to assess the relationship between changes in diabetes outcomes and ***c*^diff^**. This section proceeds with a step-by-step description of how our model evolved as we considered different facets of the data and our analytic goals.

First, it is unreasonable to assume a clinic’s average diabetes outcomes immediately benefits from its choice of clinic structure. For this reason, 2009 and 2012 outcome and covariate data from MNCM were matched with 2008 and 2011 PPCRS data to allow for a one-year lag period between policy introduction and outcome realization. Throughout, 2009 patient-level diabetes outcomes are assumed to be generated from a Gaussian distribution with clinic-specific mean and variance

(1) $$Y_{\mathrm{sji}}^{\left(2009\right)}\sim N(\mu_{\mathrm{sj}}^{\left(2009\right)},\sigma_{\mathrm{sj}}^{2}),\;s=1,\ldots ,n^{\left(s\right)},\;j=1,\ldots ,n_{s}^{\left(c\right)},\;i=1,\ldots ,n_{\mathrm{sj}}^{\left(p,2009\right)}.$$

Then, 2012 patient-level diabetes outcomes are also assumed to be generated from a Gaussian distribution with clinic-specific mean $\mu_{\mathrm{sj}}^{\left(2009\right)}$ modified by a clinic-specific *change* in means $\mu_{\mathrm{sj}}^{\text{diff}}$ from 2009 to 2012

(2) $$Y_{\mathrm{sji}}^{\left(2012\right)}\sim N(\mu_{\mathrm{sj}}^{\left(2009\right)}+\mu_{\mathrm{sj}}^{\text{diff}},\sigma_{\mathrm{sj}}^{2}),\;s=1,\ldots ,n^{\left(s\right)},\;j=1,\ldots ,n_{s}^{\left(c\right)},\;i=1,\ldots ,n_{\mathrm{sj}}^{\left(p,2012\right)},$$

where $\mu_{\mathrm{sj}}^{\text{diff}}\equiv \mu_{\mathrm{sj}}^{\left(2012\right)}-\mu_{\mathrm{sj}}^{\left(2009\right)}$ so that $E[Y_{\mathrm{sji}}^{\left(2012\right)}]=\mu_{\mathrm{sj}}^{\left(2012\right)}$. Note that 2009 and 2012 intra-clinic outcomes are assumed to share the same variance: $\text{Var}[Y_{\mathrm{sji}}^{\left(2009\right)}]=\text{Var}[Y_{\mathrm{sji}}^{\left(2012\right)}]=\sigma_{\mathrm{sj}}^{2},\;s=1,\ldots ,n^{\left(s\right)},\;j=1,\ldots ,n_{s}^{\left(c\right)},i=1,\ldots ,n_{\mathrm{sj}}^{\left(p\right)}.$

### 4.1. Continuous Difference-in-Difference Model

Our primary question of interest is answered by how the change in a clinic’s transformation (***c*^diff^**) relates to its change in mean diabetes outcome (***μ*****^diff^**). A natural model to measure this relationship is

(3) $$\mu_{\mathrm{sj}}^{\text{diff}}=\beta_{0}+\alpha c_{\mathrm{sj}}^{\text{diff}}+\epsilon_{\mathrm{sj}},\epsilon_{\mathrm{sj}}\overset{\text{iid}}{\sim }N(0,\tau^{2}),\;\text{for}\;s=1,\ldots ,n^{\left(s\right)},\;j=1,\ldots ,n_{s}^{\left(c\right)},$$

the formal statement of the CDiD model introduced in Section 1. We say “continuous” because $c_{\mathrm{sj}}^{\text{diff}}$ is a continuous treatment measure, unlike typical DiD models which feature a binary treatment indicator. The primary parameter of interest in our model is *α*, the coefficient relating change in clinic score to change in outcome.

Standard DiD estimation relies on two key assumptions: the *Common Shocks* assumption and the *Parallel Trends* assumption (Angrist and Pischke 2008). When the policy exposure is binary, the Common Shocks assumption states that external phenomena occurring at the same time or after the start of a treatment will equally affect treatment and control groups. The unadjusted analysis of Section 3 could violate this assumption: for example, urban clinics may have more exposure to other phenomena external to clinic structure that influence diabetes outcomes, for example, public health campaigns to incentivize exercise or lower sugar intake. The Parallel Trends assumption states that, absent treatment, the potential outcomes of the treatment and control groups are expected to change at the same rate. In our context with a continuous measure of exposure, the Parallel Trends assumption states that the expected rate of change in potential outcome from pre- to post-policy enactment is the same for any observed value of exposure. An unadjusted analysis could also violate this assumption: for example, clinics with a wealthier patient population may have better trends in diabetes outcomes prior to clinic restructuring, for example. To address these issues, we include clinic-year-level changes in patient demographics meant to capture external phenomena driving changes in mean diabetes outcomes. We include as covariates the change in proportion of patients with commercial insurance, the change in mean age, the change in proportion of patients that were female, the change in proportion of patients with IVD, the change in average wealth, the change in average income/education, and the change in average proportion of non-Hispanic white residents in the patients’ ZIP codes. There may also be a change in a clinic’s population that is driven at the neighborhood level. For example, gentrification may drastically change a local clinic’s patient population, biasing our estimated causal effect if left unaccounted for. A patient’s neighborhood’s ACS measured Wealth (Wlth) and Income/Education (Inc), as well as their neighborhood’s percentage of non-Hispanic white residents (NHW), can be mapped to their MNCM record. Since not all patients at a given clinic live in the clinic’s own ZIP code, we take the clinic‘s sample mean of all patients’ ZIP code Wlth, Inc, and NHW values as an estimate of a clinic’s average neighborhood characteristics.

(4) $$\mu_{\mathrm{sj}}^{\text{diff}}=X_{\mathrm{sj}}\Theta +\epsilon_{\mathrm{sj}},\epsilon_{\mathrm{sj}}\overset{\text{iid}}{\sim }N(0,\tau^{2}),\;\text{for}\;s=1,\ldots ,n^{\left(s\right)},j=1,\ldots ,n_{s}^{\left(c\right)},\;\text{where}\;X_{\mathrm{sj}}=[1,c_{\mathrm{sj}}^{\text{diff}},{\text{Comm}}_{\mathrm{sj}}^{\text{diff}},{\text{Age}}_{\mathrm{sj}}^{\text{diff}},{\text{Fem}}_{\mathrm{sj}}^{\text{diff}},{\text{IVD}}_{\mathrm{sj}}^{\text{diff}},{\text{Wlth}}_{\mathrm{sj}}^{\text{diff}},{\text{Inc}}_{\mathrm{sj}}^{\text{diff}},{\text{NHW}}_{\mathrm{sj}}^{\text{diff}}]$$

and **Θ** = [*β*_0_, *α*, *β*_1_, ..., *β*_7_]*^T^*. An equivalent multivariate representation of (4) is $\mu^{\mathrm{diff}}\sim N_{n^{\left(c\right)}}(X\Theta ,\Sigma )$, where ***X*** is the design matrix inferred from (4) and $\Sigma =\tau^{2}I_{n^{\left(c\right)}}$ where ***I**_n_* is the *n* by *n* identity matrix.

Section 4.6 further discusses our need for covariate adjustment in light of identifiability assumptions, and also presents justifications why adjusting for “post-treatment” covariates is unproblematic in this context.

### 4.2. Incorporating Error in Covariates

The CDiD model (4) assumes that its covariates, which are composed of clinic-year sample means and proportions and not patient-level data points, are fixed. However, this assumption does not capture the differing levels of variability in the mean covariates, due to differing numbers of patient observations per clinic and differing levels of patient heterogeneity per clinic. To relax this assumption, we suppose that Clinic *j* and year ^(yr)^ Commercial insurance counts ${\text{X.Comm}}_{\mathrm{sj}}^{\left(\text{yr}\right)}$, mean age ${\text{Age}}_{\mathrm{sj}}^{\left(\text{yr}\right)}$, number of females ${\text{X.Fem}}_{\mathrm{sj}}^{\left(\text{yr}\right)}$, and number of patients with IVD ${\text{X.IVD}}_{\mathrm{sj}}^{\left(\text{yr}\right)}$ arise from binomial or Gaussian distributions with clinic *j*-specific parameters where appropriate; that is, we assume ${\text{X.Comm}}_{\mathrm{sj}}^{\left(\text{yr}\right)}\sim \text{Bin}(n_{\mathrm{sj}}^{\left(\text{p,yr}\right)},\theta_{\mathrm{sj}}^{\left(\text{yr}\right)})$, ${\text{Age}}_{\mathrm{sj}}^{\left(\text{yr}\right)}\sim N(\eta_{\mathrm{sj}}^{\left(\text{yr}\right)},\sigma_{\mathrm{sj},\text{age}}^{2}∕n_{\mathrm{sj}}^{\left(\text{p,yr}\right)})$, ${\text{X.Fem}}_{\mathrm{sj}}^{\left(\text{yr}\right)}\sim \text{Bin}(n_{\mathrm{sj}}^{\left(\text{p,yr}\right)},\gamma_{\mathrm{sj}}^{\left(\text{yr}\right)})$, and ${\text{X.IVD}}_{\mathrm{sj}}^{\left(\text{yr}\right)}\sim \text{Bin}(n_{\mathrm{sj}}^{\left(\text{p,yr}\right)},\kappa_{\mathrm{sj}}^{\left(\text{yr}\right)})$, where ${\text{Comm}}_{\mathrm{sj}}^{\left(\text{yr}\right)}={\text{X.Comm}}_{\mathrm{sj}}^{\left(\text{yr}\right)}∕n_{\mathrm{sj}}^{\left(\text{p,yr}\right)}$, ${\text{Fem}}_{\mathrm{sj}}^{\left(\text{yr}\right)}={\text{X.Fem}}_{\mathrm{sj}}^{\left(\text{yr}\right)}∕n_{\mathrm{sj}}^{\left(\text{p,yr}\right)}$, and ${\text{IVD}}_{\mathrm{sj}}^{\left(\text{yr}\right)}={\text{X.IVD}}_{\mathrm{sj}}^{\left(\text{yr}\right)}∕n_{\mathrm{sj}}^{\left(\text{p,yr}\right)}$ with yr ϵ {2009, 2012}. We treat *c_sj_*, Wlth*_sj_*, Inc*_sj_*, and NHW*_sj_* as fixed, as they are not patient-level data.

### 4.3. Incorporating Health Care System Effects

Clinics within the same health care system are more similar in terms of resources, policies, and geography than clinics in different systems, so it is unreasonable to assume that clinics within the same system are uncorrelated. To explicitly model system-level dependence, the error term in the CDiD model (4) is expanded to include a system-specific fixed effect *ψ_*s*_*: *ϵ_sj_* ~ *N*(*ψ_s_*, *τ*^2^). All system-specific effects are then assumed to arise from a Gaussian distribution centered around 0: $\psi_{s}\overset{\text{iid}}{\sim }N(0,\zeta^{2}),s=1,\ldots ,n^{\left(s\right)}$.

In this framework, the clinic-level error covariance **Σ** implied by model (4) must incorporate both the variance of $\mu_{\mathrm{sj}}^{\text{diff}}$ and intra-level i correlations. Namely, **Σ** is a block diagonal matrix whose blocks correspond to systems: diagonal elements are *τ*^2^ + *ζ*^2^, off-diagonal elements within blocks are *ζ*^2^, and elements elsewhere are 0. Blocks are $n_{s}^{\left(c\right)}$ by $n_{s}^{\left(c\right)}$ square matrices.

### 4.4. Modeling Baseline Diabetes Outcomes

Sections 4.1–4.3 focused on modeling the change in outcome ***μ*****^diff^**, and not on average outcomes at baseline given in model (1). In practice, the change in clinic score and other covariates may also be associated with baseline outcomes. For example, urban clinics with more resources to commit to pursue changes needed for Minnesota health care home certification may have patients with higher baseline A1c scores. To account for these effects, we can model the baseline clinic score in a similar fashion to (4)

(5) $$\mu_{\mathrm{sj}}^{\left(2009\right)}=X_{\mathrm{sj}}\tilde{\Theta }+{\tilde{\epsilon }}_{\mathrm{sj}},{\tilde{\epsilon }}_{\mathrm{sj}}\overset{\text{iid}}{\sim }N(\upsilon_{s},\lambda^{2}),\;\text{for}\;s=1,\ldots ,n^{\left(s\right)},j=1,\ldots ,n_{s}^{\left(c\right)},$$

where $\tilde{\Theta }={\left[{\tilde{\beta }}_{0},\tilde{\alpha },{\tilde{\beta }}_{1},\ldots ,{\tilde{\beta }}_{7}\right]}^{⊺}$, with each system-specific fixed effect arising from a zero-mean Gaussian distribution: *υ_s_* ~ *N*(0, *ω*^2^). An equivalent expression of (5) is $\mu^{\left(2009\right)}\sim N_{n^{\left(c\right)}}(X\tilde{\Theta },\tilde{\Sigma })$, where $\tilde{\Sigma }$ is a block diagonal matrix whose blocks correspond to systems: with *λ*^2^ + *ω*^2^ on the diagonals, *ω*^2^ on the off-diagonals within the same block, and 0 elsewhere.

Note that in (5), we are modeling baseline ***μ***^**(2009)**^ with the same predictors (*X*) used to model change ***μ*****^diff^**. This may be counter-intuitive, especially because the change in clinic score and change in covariates given in *X* occur after baseline. However, it is critically important that ***μ***^**(2009)**^ and ***μ*****^diff^** are modeled with the same set of predictors; otherwise, coefficients may be biased if predictors included for ***μ*****^diff^** are also associated with ***μ***^**(2009)**^ or vice-versa. This phenomenon is illustrated with a simulation study in Section 8.2.

### 4.5. Baseline Clinic Score

A clinic’s trajectory in primary care transformation and mean diabetes outcomes may both be confounded by its access to resources, willingness/ability to restructure its primary care, and other factors present at the baseline year of 2008. Thus, baseline clinic scores $c_{\mathrm{sj}}^{\left(2008\right)}$ are included as fixed covariates in both models (4) and (5). Accounting for this potential source of confounding also avoids “ceiling effects”, where clinics that had already implemented many of the programs and resources measured by the PPCRS had consequently already improved diabetes outcomes, so that additional change spurred by health care home certification would be minimal. The interaction term $c_{\mathrm{sj}}^{\left(2008\right)}*c_{\mathrm{sj}}^{\mathrm{diff}}$ was investigated as a potential covariate, but ultimately excluded as it did not have a clear effect nor did it improve model fit.

### 4.6. Causal Estimand and Assumptions for Identification

Given the models in (4) and (5), our primary parameter of interest *α* resolves to $\alpha =E[Y_{\mathrm{sji}}^{\left(2012\right)}-Y_{\mathrm{sji}}^{\left(2009\right)}|c_{\mathrm{sj}}^{\text{diff}}=a+1,X_{\mathrm{sj}}^{*}=x_{\mathrm{sj}}^{*}]-E[Y_{\mathrm{sji}}^{\left(2012\right)}-Y_{\mathrm{sji}}^{\left(2009\right)}|c_{\mathrm{sj}}^{\text{diff}}=a,X_{\mathrm{sj}}^{*}=x_{\mathrm{sj}}^{*}]$, where $X_{sj}^{*}$ denotes the set of covariates ***X**_sj_*, without $c_{\mathrm{sj}}^{\text{diff}}$. Letting *Q*(*a*) denote the potential outcome of random variable *Q* had its measurement been taken at a clinic with *c*^diff^ = *a*, our causal estimand of interest here is $E[Y_{\mathrm{sji}}^{\left(2012\right)}(a+1)-Y_{\mathrm{sji}}^{\left(2012\right)}(a)]$ (Lechner 2010), the average treatment effect among the treated in the post-treatment period, where the “treatment” is clinic *j* within system *s* had increased their speed of adopting the PCMH model by one unit higher over time. In this section, we outline the assumptions under which our data can estimate this causal estimand of interest.

Model (4) conditions on “post-treatment” covariates, in that we are adjusting for the change in covariates *after* a clinic adopts PCMH policies. There is an extensive literature warning against conditioning on post-treatment covariates when attempting to estimate a causal treatment effect (Rosenbaum 1984; Montgomery, Nyhan, and Torres 2017), in the clinical or observational setting. Under the Rubin causal framework, the treatment effect is the difference in outcome between the treatment group and the “counterfactual,” or the treatment group if it had not received the treatment (Rubin 1974). If the treatment affects the covariates’ observed values, conditioning on post-treatment measurements of those covariates biases the estimated causal effect. The first assumption required to identify $E[Y_{\mathrm{sji}}^{\left(2012\right)}(a+1)-Y_{\mathrm{sji}}^{\left(2012\right)}(a)]$ is Exogeneity, which states that realized values of the covariates are not changed by the policy exposure (Lechner, 2010):

*Assumption 1* (*Exogeneity*). ***X***(*a*) = ***X***(*a′*) for any two *c*^diff^ values *a*, *a′*.

Fortunately, this assumption’s validity is not a concern in our context. There is little empirical or contextual evidence to suggest that a clinic’s speed to adopt the PCMH model affects clinic-level demographic changes; pairwise correlations between ***c*^diff^** and change in the 7 covariates considered range from $\hat{\rho }=-0.20$ to $\hat{\rho }=0.07$, with no associations corresponding to a *p*-value less than 0.01. So, the adoption of the PCMH model has a small or non-existent effect on a patient’s choice of clinic, a choice probably based more on static confounders like the patient’s geographic location and insurance type. In the absence of such exogeneity, marginal structural models (Robins, Hernan, and Brumback 2000) represent an alternative approach that can still lead to consistent effect estimates.

As alluded to in Section 4.1, the Parallel Trends assumption is necessary to identify $E[Y_{\mathrm{sji}}^{\left(2012\right)}(a+1)-Y_{\mathrm{sji}}^{\left(2012\right)}(a)]$ in DiD estimation (Abraham and Sung 2018; Athey and Imbens 2018; de Chaisemartin and D’Haultfœuille 2018). Let $Y_{\mathrm{sji}}^{\text{diff}}\equiv Y_{\mathrm{sji}}^{\left(2012\right)}-Y_{\mathrm{sji}}^{\left(2009\right)}$ denote the change in diabetes management outcome from 2009 to 2012 for a particular patient. Although $Y_{\mathrm{sji}}^{\text{diff}}$ is unobservable (recall we are unable to match patients across time) we must assume its potential outcomes are equal across observed $c_{\mathrm{sj}}^{\text{diff}}$ in expectation. We thus extend the Parallel Trends assumption, which is typically concerned with the potential outcomes of two groups defined by a binary exposure, to our context with no proper control group and a continuous exposure:

*Assumption 2* (*Parallel Trends*). $E[Y_{\mathrm{sji}}^{\text{diff}}(a)|c_{\mathrm{sj}}^{\text{diff}}=a,X_{\mathrm{sj}}^{*}=x_{\mathrm{sj}}^{*}]=E[Y_{\mathrm{sji}}^{\text{diff}}(a)|c_{\mathrm{sj}}^{\text{diff}}=a^{\prime },X_{\mathrm{sj}}^{*}=x_{\mathrm{sj}}^{*}]$, for any two *c*^diff^ values *a*, *a′*.

In other words, if the actual observed $c_{\mathrm{sj}}^{\text{diff}}$ were *a′*, and we substituted the counterfactual *a*, we would expect the same difference in outcome as if the observed value of $c_{\mathrm{sj}}^{\text{diff}}$ were *a*. While we are unable to empirically verify the appropriateness of Assumption 2, we have no contextual reasons to believe it is violated once we’ve adjusted for the covariates described in Section 4.1.

The final assumption required to identify $E[Y_{\mathrm{sji}}^{\left(2012\right)}(a+1)-Y_{\mathrm{sji}}^{\left(2012\right)}(a)]$ is the No Anticipatory Behavior assumption (Abraham and Sung 2018; Athey and Imbens 2018). Letting $Y_{\mathrm{sji}}^{\left(2009\right)}(a)$ denote the 2009 outcome for a particular patient visiting a clinic that will observe $c_{\mathrm{sj}}^{\text{diff}}=a$, the No Anticipatory Behavior assumption states that the future level of policy adoption has no effect on any two pre-policy potential outcomes:

*Assumption 3* (*No Anticipatory Behavior*). $E[Y_{\mathrm{sji}}^{\left(2009\right)}(a)-Y_{\mathrm{sji}}^{\left(2009\right)}(a^{\prime })|X_{sj}=x_{sj}]=0$, for any two *c*^diff^ values *a, a′*.

Because the legislation was passed in 2008, giving the clinics time between passage and the 2010 implementation to react, there is a risk that this assumption is not met. The measure ***c*^diff^** is captured through the PPCRS, available only in 2008 and 2011, so we cannot examine Assumption 3 formally. However, in other work currently in progress, we use binary certification dates as our treatment variable. In this work, we compare trends in outcomes for this initial wave of certified clinics with later adopters. While we see some evidence of anticipatory behavior in later adopters, the “early wave” of clinics measured in this work shows no sign of anticipatory behavior.

If Assumptions 1–3 are met, the *α* as estimated by our model identifies the average treatment effect among the treated post-policy introduction: $\alpha =E[Y_{\mathrm{sji}}^{\left(2012\right)}(a+1)-Y_{\mathrm{sji}}^{\left(2012\right)}(a)]$ (Abraham and Sung 2018; Athey and Imbens 2018; de Chaisemartin and D’Haultfœuille 2018). A proof is available in Appendix B.

## 5. Prior Specification

Here we discuss prior specification for the unknown parameters in the hierarchical likelihood model outlined in Section 4.

### 5.1. Priors for *σ*^2^ and $\sigma_{\mathrm{age}}^{2}$

Recall that the variances for the outcome (***σ*****^2^**) and patient age ($\sigma_{\mathrm{age}}^{2}$) may differ among clinics. Instead of imposing non-informative priors on each $\sigma_{\mathrm{sj}}^{2}$ and $\sigma_{\mathrm{sj},\text{age}}^{2}$, we use a data-informed empirical Bayes approach to estimate inverse-gamma priors for each. We have many $\sigma_{\mathrm{sj}}^{2}$ and $\sigma_{\mathrm{sj},\text{age}}^{2}$ parameters to estimate, and we wish to use information across all clinics to infer the distribution of these variances.

Since $\sigma_{\mathrm{sj}}^{2}$ and $\sigma_{\mathrm{sj},\text{age}}^{2}$ are “level-one” parameters that describe the variance of patient data, we can simply match the shape and scale parameters with the first two moments of the inverse-gamma distribution. Namely, for $\pi (\sigma_{\mathrm{sj}}^{2})\overset{\text{iid}}{\sim }\text{IG}(a,b),s=1,\ldots ,n^{\left(s\right)},j=1,\ldots ,n_{s}^{\left(c\right)}$, the prior mean and variance are $E[\sigma^{2}]=b∕(a-1)$ for *a* > 1 and var[*σ*^2^] = *b*^2^/[(*a*−1)^2^(*a*−2)] for *a* > 2. Solving for *a* and *b*, we obtain

(6) $$a={\left[E(\sigma^{2})\right]}^{2}∕\text{Var}(\sigma^{2})+2\;\text{and}\;b=E(\sigma^{2})\left\{{\left[E(\sigma^{2})\right]}^{2}∕\text{Var}(\sigma^{2})+1\right\}.$$

In our analysis, *a* and *b* were estimated by $\hat{a}$ and $\hat{b}$ by taking the clinic-level sample variances of the outcome ***Y***, $\sigma^{2}=[{\hat{\sigma }}_{1}^{2},\ldots ,{\hat{\sigma }}_{n^{\left(c\right)}}^{2}]$, and substituting *E*(*σ*^2^) and Var(*σ*^2^) in (6) with $\hat{E}(\sigma^{2})=(1∕n^{\left(c\right)})\sum_{j=1}^{n^{\left(c\right)}}{\hat{\sigma }}_{j}^{2}$ and $\mathrm{Var}(\sigma^{2})=(1∕(n^{\left(c\right)}-1))\sum_{j=1}^{n^{\left(c\right)}}({\hat{\sigma }}_{j}^{2}-\hat{E}(\sigma^{2}){)}^{2}$. The prior for each $\sigma_{\mathrm{sj}}^{2}$ is then inverse-gamma with shape $\hat{a}$ and scale $\hat{b}$:$\pi (\sigma_{\mathrm{sj}}^{2})=\text{IG}(\hat{a},\hat{b}),\;s=1,\ldots ,n^{\left(s\right)},j=1,\ldots ,n_{s}^{\left(c\right)}$. The same process is used to estimate the prior distribution for each$\sigma_{\mathrm{sj},\text{age}}^{2}$, using the clinic-level sample variances of patient age:$\pi (\sigma_{\mathrm{sj},\text{age}}^{2})=\text{IG}({\hat{a}}_{\text{age}},{\hat{b}}_{\text{age}}),\;s=1,\ldots ,n^{\left(s\right)},j=1,\ldots ,n_{s}^{\left(c\right)}$.

### 5.2. Remaining Prior Specification

We know little about the remaining distributions of the parameters in our model, so we use priors that are relatively uninformative. Specifically, we use flat priors for the system-level hierarchical effects and clinic-specific mean age: $\pi (\psi_{s})\propto \pi (\upsilon_{s})\propto \pi (\eta_{\mathrm{sj}}^{\left(\text{yr}\right)})\propto 1$, uniform priors for proportion parameters: $\pi (\theta_{\mathrm{sj}}^{\left(\text{yr}\right)})=\pi (\gamma_{\mathrm{sj}}^{\left(\text{yr}\right)})=\pi (\kappa_{\mathrm{sj}}^{\left(\text{yr}\right)})=U(0,1)$, and log-uniform priors for hierarchical variance parameters: *π*(*τ*^2^) ∝ 1 / *τ*^2^, *π*(*λ*^2^) ∝ 1 / *λ*^2^, *π*(*ζ*^2^), ∝ 1 / *ζ*^2^, π(*ω*^2^) ∝ 1 / *ω*^2^, for $s=1,\ldots ,n^{\left(s\right)},\;j=1,\ldots ,n_{s}^{\left(c\right)}$, yr ϵ {2009, 2012}.

## 6. Posterior Estimation

The full joint likelihood implied by the hierarchical model in Section 4 is

(7) L(μ(2009),μdiff,σ2,Θ,τ2,θ(2009),θ(2012),σage2,γ(2009),γ(2012),κ(2009),κ(2012)),η(2009),η2012,ϕ,υ,ζ2,Θ~,λ2,ω2(∣Y(2009),Y(2012),X)=∏s=1n(s){∏j=1ns(c)[∏i=1nsj(p,2009)N(Ysji(2009)∣μsj(2009),σsj2)×∏i=1nsj(p,2012)N(Ysji(2012))∣μsj(2009)+μsjdiff,σsj2)]∗N(μsjdiff∣XsjΘ+ψs,τ2)N(μsj∣XsjΘ~+υs,λ2)∗∏yr∈{2009,2012}[Bin(X.Commsj(yr)∣nsj(p,yr),θsj(yr))×N(Agesj(yr)∣ηsj(yr),σsj,age2∕nsj(p,yr))]∗Bin(X.Femsj(yr)∣nsj(p,yr),γsj(yr))Bin(X.IVDsj∣nsj(p,yr),κsj(yr))]{×N(ψs∣0,ζ2)N(υs∣0,ω2)}.

Following Lindley and Smith (1972), the distribution of **Θ**∣***μ*****^diff^** is $N_{n^{\left(c\right)}}\left(B_{1}b_{1},B_{1}\right)$, where $B_{1}^{-1}=X^{T}\Sigma^{-1}X+\Sigma_{0}^{-1}$ and $b_{1}=X^{T}\Sigma^{-1}\mu^{\text{diff}}+\Sigma_{0}^{-1}X_{0}\Theta_{0}$, where ***X*_0_Θ_0_** and **Σ_0_** are the prior mean and variance of ***μ*^diff^**, respectively. Setting $\Sigma_{0}^{-1}=0$ simplifies ***B*_1_** and ***b*_1_** to (***X^T^*Σ^−1^*X***)^−1^ and ***X^T^*Σ^−1^*μ*^diff^**, respectively, specifying a non-informative prior for ***μ*****^diff^**. The same approach is used to obtain the distribution of $\tilde{\Theta }|\mu^{\left(2009\right)}$ as$N_{n^{\left(c\right)}}\left(B_{2}b_{2},B_{2}\right)$, where $B_{2}={\left(X^{T}{\tilde{\Sigma }}^{-1}X\right)}^{-1}$ and $b_{2}=X^{T}{\tilde{\Sigma }}^{-1}\mu^{\left(2009\right)}$.

Each parameter’s full conditional can be derived from Equation (7) and the priors specified in Section 5. Posterior distributions for each parameter are then estimated with *T* = 100,000 Gibbs draws, excluding the first 10,000 draws for burn-in. Appendix A describes the Gibbs sampler in full technical detail. Convergence was verified via an assessment of trace and autocorrelation plots. RStudio Version 1.0.136 was used to perform data cleaning and analysis.

To avoid unnecessary complexity and collinearity (especially between highly correlated ACS covariates), a Deviance Information Criterion (DIC) (Spiegelhalter et al. 2002) based forward selection algorithm was used to select covariates for the models in (4) and (5). Specifically, the DIC is computed with ***X*** = [**1**, **c**^**diff**^] (since *α* is our primary measure of interest, **c**^**diff**^ must be included in the model). The reductions in DIC are computed for each predictor by adding only itself to ***X***, sorted by greatest reduction to least, added to ***X*** in order, and the algorithm stopped once the DIC is not reduced by the addition of another predictor.

## 7. Results

For the change in A1c outcome, the forward variable selection described in Section 6 yields the lowest DIC when ***X*** = [**1**, **c^diff^**, **Comm^diff^**, **Age^diff^**, **Female^diff^**, **IVD^diff^**, **c(^2008^)**], so that **Θ** = *β*_0_, *α*, *β*_1_, *β*_2_, *β*_3_, *β*_4_, *α*_0_]*^T^*. For the change in LDL and change in SBP outcomes, the algorithm yields smaller subsets of this matrix, so we use the same covariate set across outcomes for simplicity. Table 3 displays, for each diabetes outcome, 95% equal-tail Bayesian credible intervals for each coefficient in **Θ**.

The posterior credible intervals (CI) for *α* include 0 across diabetes outcomes, suggesting that change in clinic score is not significantly associated with improving diabetes outcomes after adjusting for other changes in a clinic’s patient characteristics and baseline clinic score. The change in mean age is negatively associated with A1c, suggesting that clinics whose patient pool got older on average saw a decrease in A1c score, adjusted for all other covariates. The baseline clinic score for A1c is positively significant, suggesting that clinics with greater baseline clinic score—indicating higher initial implementation of the programs and resources measured by the PPCRS—see patients whose A1c score *increased* from 2008 to 2011. A robustness test removing the baseline clinic score results in an A1c 95% CI for *α* that is significantly negative, in contrast to the results above that include 0 in the A1c 95% CI for *α*.

The two significant credible intervals in Table 3 tell a surprising story: that clinics whose patient population got *older* and that had *fewer* PCMH-related resources at baseline observed *improved* mean A1c scores from 2009 to 2012. Since our analysis is limited to patients with Type 2 diabetes, this suggests that older patients living with diabetes have a more mild form or later onset of the disease, explaining the lowering A1c levels. From a policy perspective, the significant positive signal for *α*_0_ suggests a ceiling effect of PCMH clinics—that is, those clinics which start out with many PCMH-related resources had smaller opportunity for improvement in diabetes outcomes than clinics with few PCMH-related resources. A brief analysis revealed that clinics in the top ***c***^**(2008)**^ quartile started with low mean A1c scores, and these scores increased from 2009 to 2012, while clinics in the bottom ***c***^**(2008)**^ quartile had unchanging high mean A1c scores. This suggests that clinics with more baseline access to resources serve communities more representative of the broader population that is worsening over time with respect to diabetes outcomes, while clinics with less baseline access to resources serve communities that fail to control A1c levels consistently across time.

**Figure 2.**
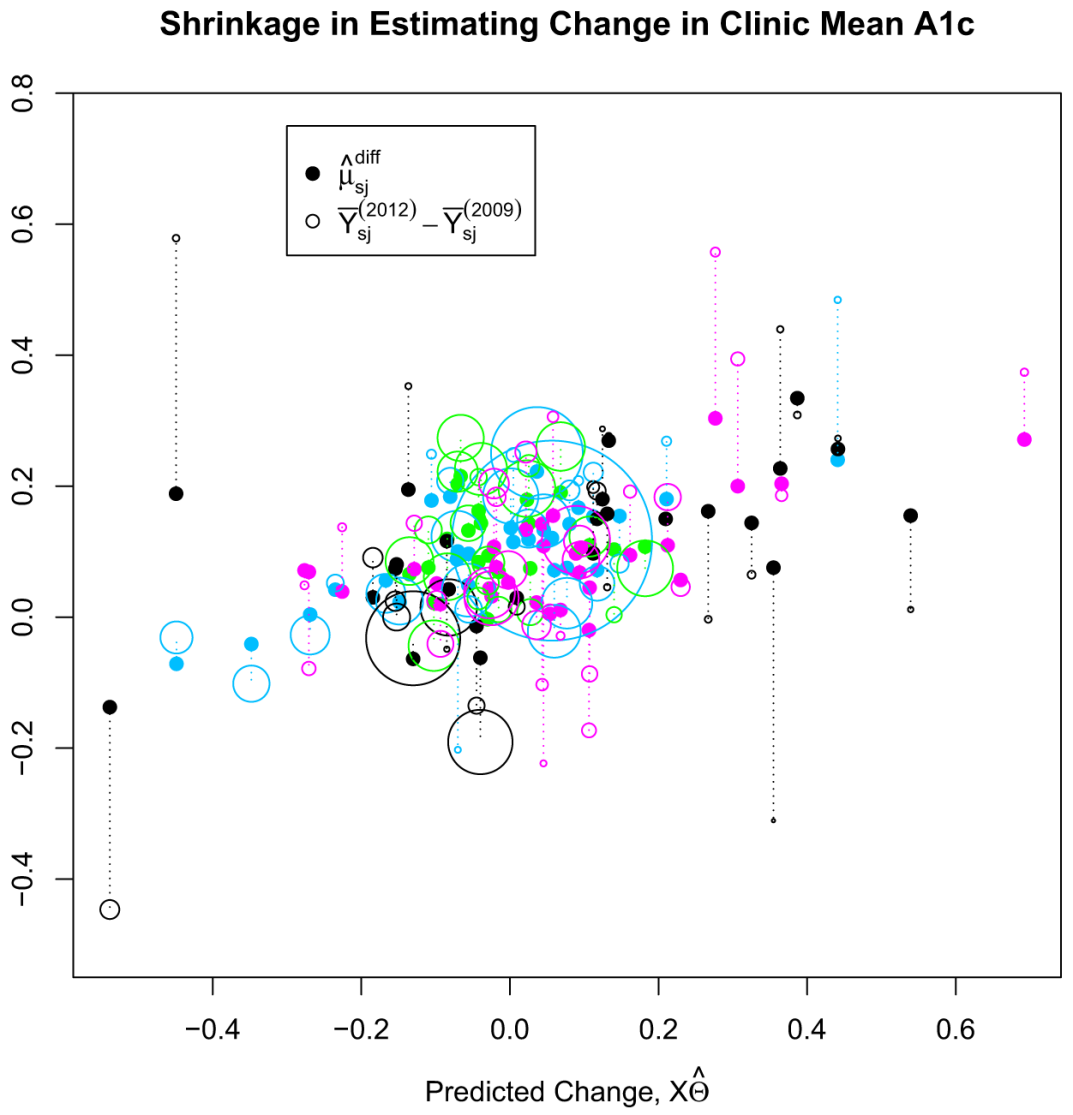
Shrinkage of clinics’ sample differences toward their predicted differences given by $X\hat{\Theta }$ for A1c, scaled by their relative sample sizes; clinics within the three largest systems are colored magenta, blue, and green.

In the ad-hoc analysis of Section 3, clinic-year sample sizes were ignored, allowing for patient outcomes at smaller clinics to influence the results more. Figure 2 illustrates how accounting for a clinic’s size and changes in covariates shrinks the sample difference in mean A1c $\left({\bar{Y}}^{\left(2012\right)}-{\bar{Y}}^{\left(2009\right)}\right)$ towards their predicted values $X\hat{\Theta }$ via their latent clinic effects ***μ*****^diff^**, where $\hat{\Theta }$ are the posterior means of **Θ**. In Figure 2, the sample difference of clinic *sj* was scaled by $1∕(1∕n_{\mathrm{sj}}^{p,2009}+1∕n_{\mathrm{sj}}^{p,2012})$, so that larger clinics appear relatively larger than smaller clinics. Closed circles correspond to the clinic’s predicted mean change ***μ*****^diff^**. Clinics belonging to the three largest systems are colored magenta, blue, and green. On average, smaller clinics are shrunk significantly towards their predicted values, while estimates of sample difference in mean A1c at larger clinics are relatively precise and shrink much less.

**Figure 3.**
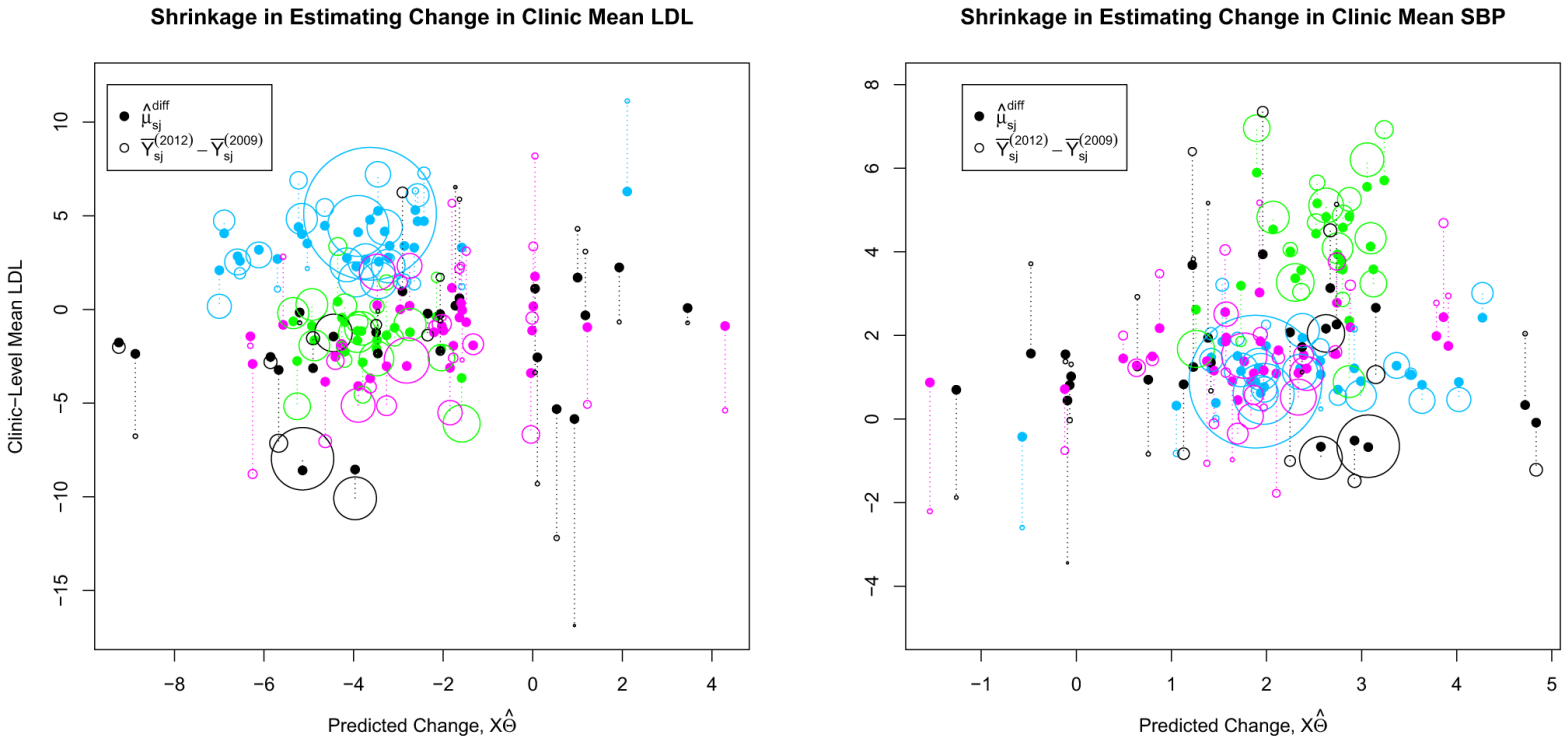
Shrinkage of clinics’ sample differences for LDL (left) and SBP (right), scaled by their relative sample sizes; clinics within the three largest systems are colored magenta, blue, and green.

Similar plots showing shrinkage for the difference in mean LDL and mean SBP are shown in Figure 3. For these outcomes the predictive model is less strong and dominated by the error terms, and thus the plots show no clear trend. However, both plots show substantial clustering at the system-level, and the primary mode of shrinkage appears to be toward the system effects rather than toward the predicted values. Analyses that ignore these system effects, such as the marginal correlation analysis in Section 3, can overestimate the effective number of observations and thus underestimate uncertainty.

## 8. Simulation Studies

### 8.1. Consequences of Ad-Hoc Analysis

Here, we present a brief simulation study outlining the consequences of applying the simple correlation analysis from Section 3 when the data truly follow a hierarchical structure, clinic-level averages are susceptible to dynamic confounding, and clinics have different sample sizes (each of which are aspects of our data, per the discussions of Section 4). In our simulation we use the actual clinic sample sizes and values of ***c***^**(2008)**^ and ***c***^**(2011)**^, and set $\tilde{\Theta }=[0\;0\;0.5{]}^{T}$, **Θ** ≡ [*α* *β*_1_ *β*_2_]^*T*^ = [0 1 0.5]*^T^*, ***X*** = [***c*^diff^*X*_1_*X*_2_**]^*T*^, where ***X*_1_** = 0.1**c^diff^** + ***ϵ*** with ***ϵ*** ~ ***N***(**0,** *I*) and ***X_2_*** ~ ***N***(**0, *I***). With these assumptions, we generate for every patient *i* within clinic *j* within system *s*, $\upsilon_{s}\sim N(0,1),\psi_{s}\sim N(0,1),\mu_{\mathrm{sj}}\sim N(X\tilde{\Theta }+\upsilon_{s},1),\;\mu_{\mathrm{sj}}^{\text{diff}}\sim N(X\Theta +\psi_{s},1),Y_{\mathrm{sji}}^{\left(2009\right)}\sim N(\mu_{\mathrm{sj}},1)$, and $Y_{\mathrm{sji}}^{\left(2012\right)}\sim N(\mu_{\mathrm{sj}}+\mu_{\mathrm{sj}}^{\text{diff}},1)$. With each data generation, we compute $\hat{\rho }({\bar{Y}}^{\left(2012\right)}-{\bar{Y}}^{\left(2009\right)},c^{\text{diff}})$ and the p-value from a hypothesis test of *H*_0_ : *ρ* = 0 versus *H*_1_ : *ρ* ≠ 0.

With 1000 simulations, the simple correlation analysis rejected the null hypothesis 99% of the time, with an average $\hat{\rho }$ of 0.35. The 95% credible intervals computed using our proposed Bayesian hierarchical DiD model failed to capture the true value of *α* = 0 the (approximately) expected 5.1% of the time. The results here and those in Section 3 show that an unadjusted correlational analysis can suggest an effect where none truly exists, and should serve as a cautionary tale to investigators using DiD models to estimate causal effects where data have a hierarchical structure and dynamic confounders.

### 8.2. Consequences of Omitting Important Predictors at Baseline

The reader still may wonder why ***μ*****^(2009)^** and ***μ*****^diff^** must be modeled using the same predictor set (see Section 4.4). Our primary interest is in modeling change (***μ*****^diff^**), and because most of our predictors are change to a point in time after baseline it may be counter-intuitive to use them as predictors for mean at baseline, ***μ*****^(2009)^**. However, even if the predictive model for ***μ*****^diff^** (4) is appropriately specified, coefficient estimates may still be biased if predictors correlate with ***μ*****^(2009)^** but are not included in a linear model for baseline (5). We conduct a simulation study to illustrate this by comparing the bias incurred when estimating **Θ** and $\tilde{\Theta }$ under two different mean structures: $E[\mu^{\left(2009\right)}|\mu_{0}]=\mu_{0}1$(MS1) and $E[\mu^{\left(2009\right)}|X]=X\tilde{\Theta }$ (MS2), where **1** is a vector of 1’s.

First, covariates ***X*_1_**, ***X*_2_**, ***X*_3_**, and***X*_4_** are generated from independent and identically distributed multivariate normal distributions: $X_{1},X_{2},X_{3},X_{4}\overset{\mathrm{iid}}{\sim }(0,I_{J})$, so that ***X*** = [***X*_1_**, ***X*_2_**, ***X*_3_**, ***X*_4_**] and J is the total number of clinics. We then generate ***μ*****^(2009)^** and ***μ*****^diff^** according to multivariate normal distributions with uncorrelated errors: $\mu^{\left(2009\right)}\sim N_{J}(X\tilde{\Theta },I_{J})$ and ***μ***^**diff**^ ~ ***N***_*J*_(***X*****Θ**, ***I***_*J*_), where **θ** = [*β*_0_, *α*, *β*_1_, *β*_2_, *β*_3_]*^T^* = [0, 0, 0.5, 1, 0]*^T^* and $\tilde{\Theta }=[0,1,0.5,0,0{]}^{T}$. Thus, some covariates are predictive of only ***μ***^**(2009)**^, some are predictive of only ***μ***(**^diff^**), and some are predictive of both. Individual level outcome data for years 2009 and 2012 are then generated from Gaussian distributions similar to the model described in Section 4: $Y_{\mathrm{ji}}^{\left(2009\right)}\sim N(\mu_{j}^{\left(2009\right)},1)$ and $Y_{\mathrm{ji}}^{\left(2012\right)}\sim N(\mu_{j}^{\left(2009\right)}+\mu_{j}^{\text{diff}},1)$, for patients *i* = 1, ..., *I* = 10 at clinics *j* = 1, …, *J* = 100. System organization effects were ignored for simplicity. In both scenarios, ***μ*****^diff^** was modeled as a Gaussian with mean ***X*Θ** : ***μ*****^diff^** ~ ***N**_J_*(***X*Θ**, ***I**_J_*). We modeled ***μ***^**(2009)**^ under two different approaches: as a random effect with no covariates, ***μ***(**^2009^**) ~ ***N**_J_*(*μ*_0_**1***_J_*, ***I**_J_*) (MS1), and another with the same predictors used in the model for ***μ*****^diff^**, $\mu^{\left(2009\right)}\sim N_{J}(X\tilde{\Theta },I_{J})$ (MS2).

The simulation was run 10,000 times, each using 10,000 Gibbs draws excluding the first 1,000 for burn-in. Each parameter was initialized to its true value, and convergence was confirmed via trace plots. The full conditionals are simple to derive, so the Gibbs algorithm is omitted from the text.

Table 4 displays the biases incurred in estimating the coefficients **Θ**, to predict ***μ*****^diff^**, under models MS1 or MS2 for ***μ***(**^2009^**). Precisely those nonzero coefficients that MS1 does not account for lead to substantial bias: $\hat{\text{Bias}}(\hat{\alpha })=0.089$ and $\hat{\text{Bias}}({\hat{\beta }}_{1})=0.046$. Zero coefficients in $\tilde{\Theta }$ incur virtually no bias, which is sensible since the columns of ***X*** are independent and MS1 does not include ***X*_3_** and ***X*_4_**. Using MS2 incurs virtually no bias in estimating **Θ**, even though the model for ***μ*****^diff^** was the same, illustrating that ***μ*****^(2009)^** must share the same predictors to avoid biased estimates of *α*.

## 9. Discussion, Limitations, and Future Directions

In this article we identified one joint component across all question domains for the 2008 and 2011 PPCRS data, and considered whether a change in this component was causally associated with a change in diabetes outcomes. Although we did not find strong evidence of an effect for this component, it is likely an over-simplified measure of PCMH-related programs and resources at the clinic level. We stress that there still may be specific question domains, and policy changes more generally, that are causally relevant to diabetes outcomes. We believe that analyses of later PPCRS data will uncover more specific factors as important components that are relevant to diabetes care. In fact, a recent principal components analysis of the PPCRS sent out to a broader sample of clinics in 2017 shows a much richer structure, where up to ten groups of questions arise as important sources of survey answer variation. This motivates future work to uncover which factors are most important in diabetes care in the primary care setting, as panel data emerge for this larger sample.

Improvement in A1c should not really be measured linearly across its entire scale; for example, a patient A1c level less than 6.5% may be considered satisfactory and not in need of further reduction. However, we are aggregating patient-year level A1c scores to a clinic-year mean (each with at least 30 patient-years), and the difference in means across should still provide a reasonable summary of clinic-level A1c improvement. Encryption of patient identifiers was not consistent across years, so any inference about the effectiveness of the clinic transformation was not possible at the patient level. Our clinic-level data clearly lack precision. Also, there is a potential for recall bias in the PPCRS survey data, since the survey was implemented in 2011 and responses for 2008 were based on recall. All $c_{\mathrm{sj}}^{\text{diff}}$ were nonnegative except for one, evidence that improved programs and resources measured from 2008 to 2011 may be artificially created by PPCRS respondents’ recall. In fact, a preliminary analysis of PPCRS data from 2011 and 2017 shows that some clinics in fact regress in maturity of primary care transformation, further evidence that recall bias may be present in the 2008 to 2011 analysis. Future work will investigate which *particular* items of PPCRS are related to the biggest changes in diabetes outcomes.

Our model assumes that each patient visiting a clinic whose $c_{\mathrm{sj}}^{\text{diff}}$ value had been one unit higher benefits by a single value (*α*). We could relax this assumption by developing our model within a more general “Changes-in-Changes” (CiC) model framework, which assumes the potential outcomes are functions *h*(*u, t*) increasing monotonically in a patient’s unobservable characteristics *u*. The CiC model is much more general than the DiD model, as it allows for differential treatment effects across individuals and groups, has identifying assumptions which are invariant to monotone transformations, and allows for discrete outcomes and more than two timepoints, all while enjoying asymptotic normality and consistency properties under mild conditions (Athey and Imbens 2006).

To test the sensitivity of our findings to the prior specification, we reran the analysis with the following priors: (i) quadrupled the variance while keeping the mean constant within each $\sigma_{\mathrm{sj}}^{2}$ and $\sigma_{\mathrm{sj},\text{age}}^{2}$ inverse-gamma prior, (ii) a Jeffreys’ prior (Jeffreys 1946) for each proportion parameter: $\pi (\theta_{\mathrm{sj}}^{\left(\text{yr}\right)})=\pi (\gamma_{\mathrm{sj}}^{\left(\text{yr}\right)})=\pi (\kappa_{\mathrm{sj}}^{\left(\text{yr}\right)})=\mathrm{Beta}(1∕2,1∕2)$, and (iii) the remaining variance parameters: $\pi (\tau^{2})\propto 1∕\sqrt{\tau^{2}},\pi (\lambda^{2})\propto 1∕\sqrt{\lambda^{2}},\pi (\zeta^{2})\propto 1∕\sqrt{\zeta^{2}},\pi (\omega^{2})\propto 1∕\sqrt{\omega^{2}}$. We found that the final results yielded by using these priors differ very little from those in Table 3.

The discrepancy between the results of the analysis in Section 3 and Table 3 should serve as a cautionary tale for investigators. Failing to account for confounding and all sources of variability—system structure, covariates and their variability, baseline clinic score—would lead an investigator to falsely conclude a clear negative association between trends in A1c score and ***c***^**diff**^.

## Supplementary Material

Supp 1

Supp 2

Supp 3

## Figures and Tables

**Table 1.** Notation reference, Greek letters.

Symbol	Meaning
*μ*^(2009)^	Mean diabetes outcome, 2009
*μ*^diff^	Mean change in diabetes outcome from 2009 to 2012
*σ*^2^	Variance of diabetes outcome in both 2009 and 2012
*α*	Effect of clinic structure on mean change in diabetes outcome
Θ	Collection of coefficients described in Section 4.1
$\tilde{\Theta }$	Collection of coefficients described in Section 4.4
*ψ*	Health care system fixed effect for change in mean diabetes outcome
*υ*	Health care system fixed effect for mean diabetes outcome, 2009
*λ*^2^	Variance of error term for regression on *μ*
*τ*^2^	Variance of error term for regression on *μ*^diff^
$\sigma_{\text{age}}^{2}$	Variance of patient age
*θ*	True proportion of patients on Commercial insurance
*η*	True mean age of patients
*γ*	True proportion of female patients
*κ*	True proportion of patients with ischemic vascular disease (IVD)
*ζ*^2^	Variance of system effect *ψ*
*ω*^2^	Variance of system effect *υ*
*π*()	Prior distribution

**Table 2.** Notation reference, Roman letters.

Symbol	Meaning
(yr)	Value is specific to year yr
~	Value is specific to year 2009
sji	Value is specific to patient *i* at clinic *j* within system s
*c*	“Clinic score” measuring maturity in primary care transformation
n^(*s*)^	Total number of health care systems
n^(*c*)^	Total number of clinics
$n_{s}^{\left(c\right)}$	Total number of clinics within system *s*
$n_{\mathrm{sj}}^{\left(p\right)}$	Total number of patients at clinic *j* within system *s*
*c*^diff^	Change in clinic score from 2008 to 2011
*Y_sji_*	Patient-level diabetes outcome
Comm*_sj_*	Sample proportion of patients using commercial insurance
Age*_sj_*	Sample mean patient age
Fem*_sj_*	Sample proportion of patients who are female
IVD*_sj_*	Sample proportion of patients with ischemic vascular disease (IVD)
Wlth*_sj_*	Mean of clinic *j*’s patients’ neighborhood-level measure of wealth
Inc*_sj_*	Mean of clinic *j*’s patients’ neighborhood-level measure of income and education
NHW*_sj_*	Mean of clinic *j*’s patients’ neighborhood-level proportion of non-Hispanic
	white residents
*Q*(*a*)	Potential outcome of *Q* had it been measured at a clinic with *c*^diff^ = *a*

**Table 3.** 95% credible intervals for coefficients **Θ**.

	Change in A1c	Change in LDL	Change in SBP
Change in clinic score, *α*	(−0.023, 0.013)	(−0.312, 0.558)	(−0.244, 0.186)
Change in proportion on commercial, *β*_1_	(−0.157, 0.239)	(−3.885, 7.518)	(−1.711, 4.442)
Change in mean age, *β*_2_	**(−0.314, −0.043)**	(−6.385, 1.990)	(−0.997, 3.058)
Change in proportion female, *β*_3_	(−0.199, 0.310)	(−4.708, 9.848)	(−3.711, 4.224)
Change in proportion with IVD, *β*_4_	(−0.315, 0.399)	(−10.169, 8.293)	(−6.290, 3.864)
Baseline clinic score, *α*_0_	**(0.006, 0.031)**	(−0.186, 0.435)	(−0.251, 0.059)

**Table 4.** Estimated Biases (Bias) of **Θ** under different mean structures (MS) for ***μ***^**(2009)**^.

	$\text{Bias}(\hat{\alpha })$	$\text{Bias}({\hat{\beta }}_{1})$	$\text{Bias}({\hat{\beta }}_{2})$	$\text{Bias}({\hat{\beta }}_{3})$
MS1	+0.089	+0.046	−0.002	+0.002
MS2	−0.002	+0.000	−0.002	+0.002

## Appendix A

The following algorithm outlines the Gibbs sampler described in Section 6:

## Appendix B

Throughout this section, let (*a*) denote the potential outcome of the preceding random variable had the measurement been taken at a clinic with *c*^diff^ = *a*, and let $X_{\mathrm{sj}}^{*}$ denote the covariate set for clinic *j* within system *s*, excluding $c_{\mathrm{sj}}^{\text{diff}}$. Below we present a proof that, under Assumptions 1-3 described in Section 4.6, the *α* as specified by our model, $E[Y_{\mathrm{sji}}^{\left(2012\right)}-Y_{\mathrm{sji}}^{\left(2009\right)}|X_{\mathrm{sj}}^{*}=x_{\mathrm{sj}}^{*},c_{\mathrm{sj}}^{\text{diff}}=a+1]-E[Y_{\mathrm{sji}}^{\left(2012\right)}-Y_{\mathrm{sji}}^{\left(2009\right)}|X_{\mathrm{sj}}^{*}=x_{\mathrm{sj}}^{*},c_{\mathrm{sj}}^{\text{diff}}=a]$, identifies the estimand $E[Y_{\mathrm{sji}}^{\left(2012\right)}(a+1)-Y_{\mathrm{sji}}^{\left(2012\right)}(a)]$.

$$\alpha =E[Y_{\mathrm{sji}}^{\left(2012\right)}-Y_{\mathrm{sji}}^{\left(2009\right)}|X_{\mathrm{sj}}^{*}=x_{\mathrm{sj}}^{*},c_{\mathrm{sj}}^{\text{diff}}=a+1]-E[Y_{\mathrm{sji}}^{\left(2012\right)}\;\;-Y_{\mathrm{sji}}^{\left(2009\right)}|X_{\mathrm{sj}}^{*}=x_{\mathrm{sj}}^{*},c_{\mathrm{sj}}^{\text{diff}}=a]\;=E[Y_{\mathrm{sji}}^{\left(2012\right)}-Y_{\mathrm{sji}}^{\left(2009\right)}|X_{\mathrm{sj}}^{*}=x_{\mathrm{sj}}^{*}(a+1),c_{\mathrm{sj}}^{\text{diff}}=a+1]\;\;-E[Y_{\mathrm{sji}}^{\left(2012\right)}-Y_{\mathrm{sji}}^{\left(2009\right)}|X_{\mathrm{sj}}^{*}=x_{\mathrm{sj}}^{*}(a),c_{\mathrm{sj}}^{\text{diff}}=a]\;\;\text{by Assumption}\;1\;=E[Y_{\mathrm{sji}}^{\left(2012\right)}(a+1)-Y_{\mathrm{sji}}^{\left(2009\right)}(a+1)|X_{\mathrm{sj}}^{*}=x_{\mathrm{sj}}^{*}(a+1),c_{\mathrm{sj}}^{\text{diff}}=a+1]\;\;-E[Y_{\mathrm{sji}}^{\left(2012\right)}(a+1)-Y_{\mathrm{sji}}^{\left(2009\right)}(a+1)|X_{\mathrm{sj}}^{*}=x_{\mathrm{sj}}^{*}(a+1),c_{\mathrm{sj}}^{\text{diff}}=a+1]\;=E[Y_{\mathrm{sji}}^{\left(2012\right)}(a+1)-Y_{\mathrm{sji}}^{\left(2009\right)}(a+1)|X_{\mathrm{sj}}^{*}=x,c_{\mathrm{sj}}^{\text{diff}}=a+1]\;\;-E[Y_{\mathrm{sji}}^{\left(2012\right)}(a)-Y_{\mathrm{sji}}^{\left(2009\right)}(a)|X_{\mathrm{sj}}^{*}=x,c_{\mathrm{sj}}^{\text{diff}}=a]\;\;\text{by Assumption}\;1\;=E[Y_{\mathrm{sji}}^{\left(2012\right)}(a+1)-Y_{\mathrm{sji}}^{\left(2009\right)}(a+1)|X_{\mathrm{sj}}^{*}=x,c_{\mathrm{sj}}^{\text{diff}}=a+1]\;\;-E[Y_{\mathrm{sji}}^{\left(2012\right)}(a+1)-Y_{\mathrm{sji}}^{\left(2009\right)}(a+1)|X_{\mathrm{sj}}^{*}=x,c_{\mathrm{sj}}^{\text{diff}}=a]\;\;+E[Y_{\mathrm{sji}}^{\left(2012\right)}(a+1)-Y_{\mathrm{sji}}^{\left(2009\right)}(a+1)|X_{\mathrm{sj}}^{*}=x,c_{\mathrm{sj}}^{\text{diff}}=a]\;\;-E[Y_{\mathrm{sji}}^{\left(2012\right)}(a+1)-Y_{\mathrm{sji}}^{\left(2009\right)}(a+1)|X_{\mathrm{sj}}^{*}=x,c_{\mathrm{sj}}^{\text{diff}}=a]\;=E[Y_{\mathrm{sji}}^{\left(2012\right)}(a+1)-Y_{\mathrm{sji}}^{\left(2009\right)}(a+1)|X_{\mathrm{sj}}^{*}=x,c_{\mathrm{sj}}^{\text{diff}}=a]\;\;-E[Y_{\mathrm{sji}}^{\left(2012\right)}(a)-Y_{\mathrm{sji}}^{\left(2009\right)}(a)|X_{\mathrm{sj}}^{*}=x,c_{\mathrm{sj}}^{\text{diff}}=a]\;\;\text{by Assumption}\;2\;=E[Y_{\mathrm{sji}}^{\left(2012\right)}(a+1)-Y_{\mathrm{sji}}^{\left(2012\right)}(a)+Y_{\mathrm{sji}}^{\left(2009\right)}(a)-Y_{\mathrm{sji}}^{\left(2009\right)}(a+1)|\;\;X_{\mathrm{sj}}^{*}=x,c_{\mathrm{sj}}^{\text{diff}}=a]\;=E[Y_{\mathrm{sji}}^{\left(2012\right)}(a+1)-Y_{\mathrm{sji}}^{\left(2012\right)}(a)|X_{\mathrm{sj}}^{*}=x,c_{\mathrm{sj}}^{\text{diff}}=a]\text{by Assumption}\;3$$

So, under the assumptions of our statistical model, $\alpha =E[Y_{\mathrm{sji}}^{\left(2012\right)}(a+1)-Y_{\mathrm{sji}}^{\left(2012\right)}(a)|X_{\mathrm{sj}}^{*}=x,c_{\mathrm{sj}}^{\text{diff}}=a]\forall \{x,a\}$ Finally,

$$\alpha =E_{x}[\alpha ]\;=E\{E_{x}[Y_{\mathrm{sji}}^{\left(2012\right)}(a+1)-Y_{\mathrm{sji}}^{\left(2012\right)}(a)|X_{\mathrm{sj}}^{*}=x,c_{\mathrm{sj}}^{\text{diff}}=a]\}\;=E[Y_{\mathrm{sji}}^{\left(2012\right)}(a+1)-Y_{\mathrm{sji}}^{\left(2012\right)}(a)].$$
